# STK24 Promotes Progression of LUAD and Modulates the Immune Microenvironment

**DOI:** 10.1155/2023/8646088

**Published:** 2023-05-04

**Authors:** Yadong Li, Yanhu Liu, Kun Wang, Dong Xue, Yiqin Huang, Zhenguo Tan, Yijiang Chen

**Affiliations:** ^1^Department of Thoracic and Cardiovascular Surgery, The First Affiliated Hospital of Nanjing Medical University, Nanjing, China; ^2^Department of Thoracic and Cardiovascular Surgery, The Second Affiliated Hospital of Nanjing Medical University, Nanjing, China; ^3^The Affiliated Anning First People's Hospital, Kunming University of Science and Technology, Kunming, China

## Abstract

**Objective:**

Recent studies have shown that serine/threonine-protein kinase 24 (STK24) plays an important role in cancer development. However, the significance of STK24 in lung adenocarcinoma (LUAD) remains to be determined. This study is aimed at investigating the significance of STK24 in LUAD.

**Methods:**

STK24 was silenced and overexpressed by siRNAs and lentivirus, respectively. Cellular function was assessed by CCK8, colony formation, transwell, apoptosis, and cell cycle. mRNA and protein abundance was checked by qRT-PCR and WB assay, respectively. Luciferase reporter activity was evaluated to examine the regulation of KLF5 on STK24. Various public databases and tools were applied to investigate the immune function and clinical significance of STK24 in LUAD.

**Results:**

We found that STK24 was overexpressed in lung adenocarcinoma (LUAD) tissues. High expression of STK24 predicted poor survival of LUAD patients. In vitro, STK24 enhanced the proliferation and colony growth ability of A549 and H1299 cells. STK24 knockdown induced apoptosis and cell cycle arrest at G0/G1 phase. Furthermore, Krüppel-like factor 5 (KLF5) activated STK24 in lung cancer cells and tissues. Enhanced lung cancer cell growth and migration triggered by KLF5 could be reversed by silencing of STK24. Finally, the bioinformatics results showed that STK24 may be involved in the regulation of the immunoregulatory process of LUAD.

**Conclusion:**

KLF5 upregulation of STK24 contributes to cell proliferation and migration in LUAD. Moreover, STK24 may participate in the immunomodulatory process of LUAD. Targeting KLF5/STK24 axis may be a potential therapeutic strategy for LUAD.

## 1. Introduction

Lung adenocarcinoma (LUAD) is one of the common malignant tumors in China [1]. During the past decades, a large amount of efforts, including whole genome sequencing, RNA sequencing, and proteomics, have been made to dissect the molecular drivers for this deadly malignancy. Genetic alterations, such as EGFR-activating mutations, are identified as the essential promoter of lung cancer development [2]. Lung cancer patients harboring EGFR activation benefit from the targeted therapies of gefitinib, a specific EGFR inhibitor [3]. However, there are still some of the patients exhibiting no effectiveness when using gefitinib. Therefore, novel drug targets triggering lung cancer are constantly in need.

In recent years, immunotherapy based on immune checkpoint inhibitors (ICIs) has gradually become the focus of cancer treatment. To date, a variety of ICIs have been applied in the treatment of LUAD patients [4]. However, only a minority of patients benefit from immunotherapy [5]. Numerous evidences indicate that the leukocyte infiltration status within the tumor immune microenvironment is closely related to the response to immunotherapy [6, 7]. Therefore, the exploration and identification of novel LUAD immune-related genes are crucial for the development of LUAD treatment strategies.

STK24, which is also named as MST3, is one of the members of the mammalian sterile twenty (MST) kinase family of proteins [8]. The role of STK24 in cancers is a limited report. While STK24 plays an oncogenic role in gastric cancer growth [9], it can serve as a tumor suppressor in colorectal cancer [10]. STK24 also contributes to breast cancer development by regulating VAV2/Rac1 signaling cascade [11]. Nevertheless, the significance of STK24 in LUAD growth and migration is poorly elucidated.

Herein, we explored the role of STK24 in LUAD by analyzing its clinical significance based on TCGA database, by exploring its function on LUAD cell proliferation, cell cycle, apoptosis, migration, and immunoregulatory.

## 2. Materials and Methods

### 2.1. Cell Lines and Regents

A549 and H1299 cells were obtained from American Type Culture Collection (Manassas, USA). Dulbecco's modified eagle (DMEM), 1640 cell culture medium, and antibiotics were from Corning. Fetal bovine serum (FBS) was purchased from Gibco (California, USA). Antibodies against KLF5, STK24, *β*-actin, and all of the secondary antibodies were from Proteintech (Wuhan, China). siRNAs against negative control, STK24, and KLF5 were obtained from GenePharma (Shanghai, China). TRIzol reagent was from Invitrogen (Carlsbad, USA). The RT-for-PCR kit was from Clontech. SYBR Green qPCR mix was from Takara (Japan). Protease and phosphatase inhibitors were purchased from Roche (Basel, Switzerland).

### 2.2. Bioinformatic Analyses

In this study, we used multiple public databases and tools to investigate the biological function of STK24 in LUAD. A total of 515 cancer samples and 59 normal samples were downloaded from TCGA database. The expression of STK24 and the correlation between STK24 and PCNA, between STK24 and KLF5, and survival data were analyzed from TCGA-LUAD cohort. For survival analysis, LUAD patients were cut off by quartile.

Tumor Immune Estimation Resource 2.0 (TIMER2.0) is a web service database that can be used to systematically analyze immune cell infiltration in various cancers. This database provides a variety of analytical functions, including gene, survival, SCNA, Diff Exp, correlation, and estimation to analyze tumor immune function [12]. In the present study, we analyzed the relationship between STK24 and immune cells by somatic copy number variation (SCNV).

In this study, we applied the Cell type Identification by Estimating Relative Subsets of RNA Transcripts (CIBERSORT) algorithm to analyze the relationship between STK24 and immune cells [13]. The method relies on a matrix file called LM22 to analyze immune cells in tissues.

Tumor Immune Dysfunction and Exclusion (TIDE) is an algorithm for evaluating tumor immune escape potential via gene expression profiling in cancer samples [14]. We analyzed the relationship of STK24 and T cell dysfunction and potential regulators of tumor immune escape by this web tool.

### 2.3. Cell Culture

A549 and H1299 cells were cultured in DMEM culture medium, which contained 10% FBS and 1% antibiotics. All cells were cultured in a 37°C incubator with the constant CO_2_.

### 2.4. Real-Time Quantitative Polymerase Chain Reaction (RT-qPCR)

Lung cancer cells were lased in TRIzol, and RNA was extracted from the cells based on the manufacturer's protocols. mRNA was reversely transcribed into cDNA by using RT-for-PCR kit. Detection of indicated cDNA level was performed by using SYBR Green qPCR mix. The primer sequences were as follows: STK24 forward, 5′-AGGCATTGACAATCGGACTCA-3′, and reverse, 5′-CTGACTCAGCACTGTGATTTCT-3′. *β*-actin forward, 5′-GAGCTGCGTGTGGCTCCC-3′, and reverse, 5′-CCAGAGGCGTACAGGGATAGCA-3′.

### 2.5. Immunoblotting

Cells were lased in lysis buffer, and protein amount was detected by using BCA kit. After being boiled, proteins with loading buffer were loaded onto SDS-PAGE gels. After 1-2 hours of separation, the proteins on gels were transferred onto PVDF membranes, which were activated by methanol. After blocking with 5% skim milk and incubating with primary and secondary antibodies, protein expression was detected by using chemiluminescence reagent.

### 2.6. Cell Proliferation

CCK8 kit was used to investigate cell proliferation. At indicated time after seeding lung cancer cells in 96-well plates, 10% of CCK8 regent was added into each well, and the plates were maintained at 37°C for 3-4 hours. OD450 was then checked, and cell proliferation was normalized to day 1.

### 2.7. Colony Formation

A549 and H1299 were seeded at the concentration of 1000 cells per well. 8-12 days later, colonies were fixed by methanol and viewed by crystal violet.

### 2.8. Transwell Assay

Cell migration was examined by transwell assay. 30000 of A549 cells and 4000 of H1299 cells in 200 ul DMEM medium without FBS were plated onto the upper surface of the transwell chamber. 24 hours later, cells attached on the lower surface of the transwell chamber were fixed by methanol and viewed by crystal violet.

### 2.9. Cell Cycle

Cell cycle was detected by staining the cells with PI. Cells were washed with PBS and incubated with 70% iced alcohol overnight. Then, the cells were stained with PI, and the cell cycle was analyzed on flow cytometry.

### 2.10. Apoptosis

Trypsin without EDTA was used to trypsinize the cells when analyzing cell apoptosis. Then, the cells were stained with PI and annexin V, and apoptosis was analyzed on flow cytometry.

### 2.11. Dual Luciferase Reporter Activity

The promoter sequence of STK24 was inserted into pGL3.basic vectors. The CDS sequence of KLF5 was cloned into pCDNA3.1 vectors. After cotransfecting expressing vectors, luciferase pGL3.basic vectors, and internal control pCMV-RL-TK vectors into A549 cells, the dual luciferase activity was assessed. Luciferase activity was normalized to TK activity.

### 2.12. Statistical Analysis

Statistical data were analyzed using GraphPad Prism software. Student's *t*-test was applied to analyze the difference between the two groups. *p* < 0.05 was considered statistically significant.

## 3. Results

### 3.1. STK24 Is Overexpressed in LUAD Patients

**Figure 1:**
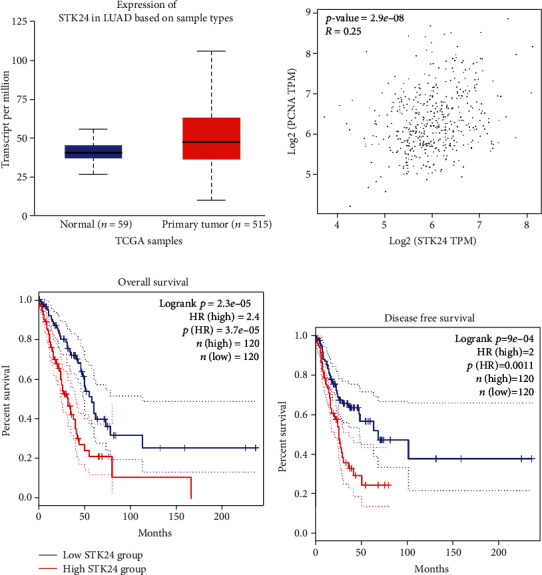
STK24 overexpression confers poor prognosis of LUAD patients. (a) Analysis of STK24 transcript in LUAD (*n* = 515) and normal tissues (*n* = 59). (b) Spearman correlation between STK24 and PCNA. (c, d) Overall and disease-free survival of LUAD patients who were divided into STK24 high expression (*n* = 120) and low expression (*n* = 120) groups. *p* < 0.01.

We initially analyzed the expression of STK24 in LUAD patients based on the public TCGA database. STK24 was upregulated in LUAD samples compared with normal tissues (Figure 1(a)). Spearman association analysis found that STK24 was positively correlated with PCNA (Figure 1(b)). Then, we analyzed the survival of LUAD patients who were divided into STK24 high-expression and low-expression groups. Both overall and disease-free survival of LUAD patients who had high expression of STK24 were shorter than that in STK24 lowly expressed patients (Figures 1(c) and 1(d)). These results strongly suggested that STK24 and LUAD are closely related to prognosis.

### 3.2. STK24 Plays a Pivotal Role in the Proliferation of LUAD

**Figure 2:**
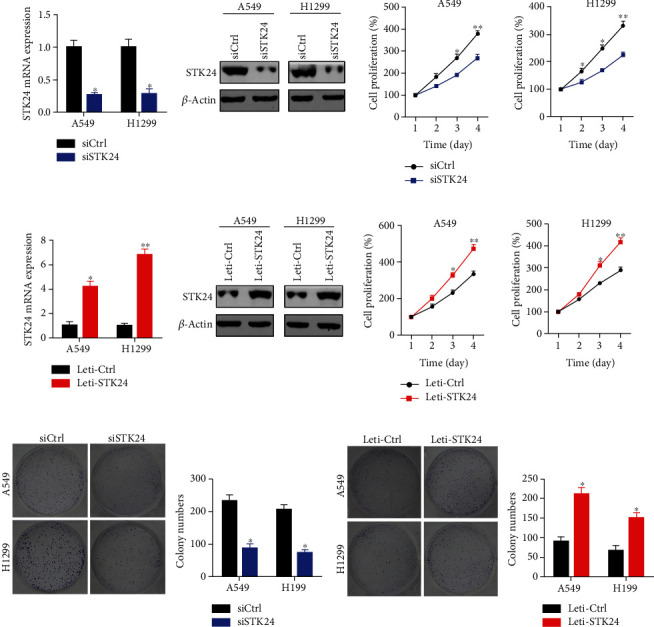
STK24 accelerates LUAD cell proliferation. (a) siCtrl and siSTK24 A549 and H1299 cells were subjected to qRT-PCR detection of STK24 mRNA level, immunoblotting detection of STK24 protein abundance, and CCK8 analysis of cell viability. (b) Leti-Ctrl and Leti-STK24 A549 and H1299 cells were subjected to qRT-PCR detection of STK24 mRNA level, immunoblotting detection of STK24 protein abundance, and CCK8 analysis of cell viability. (c, d) Colony growth was assessed. ^∗^*p* < 0.05. ^∗∗^*p* < 0.01.

To explore the function of STK24, we silenced STK24 in A549 and H1299 cells. STK24 mRNA and protein expression were efficiently reduced by siRNAs (Figure 2(a)). STK24 downregulation led to reduced proliferation of A549 and H1299 cells (Figure 2(a)). Furthermore, STK24 was upregulated after incubating with Leti-STK24 for 48 hours (Figure 2(b)). Cell proliferation ability was enhanced after STK24 ectopic expression in A549 and H1299 cells (Figure 2(b)). Colony formation results showed that STK24 downregulation suppressed the colony growth in A549 and H1299 cells (Figure 2(c)). On the contrary, STK24 overexpression potentiated the proliferation and growth ability of both cells (Figure 2(d)). Thus, our findings suggest that STK24 has an oncogenic function for lung cancer cell proliferation and growth.

### 3.3. Downregulation of STK24 Induces LUAD Apoptosis and Cell Cycle Arrest

**Figure 3:**
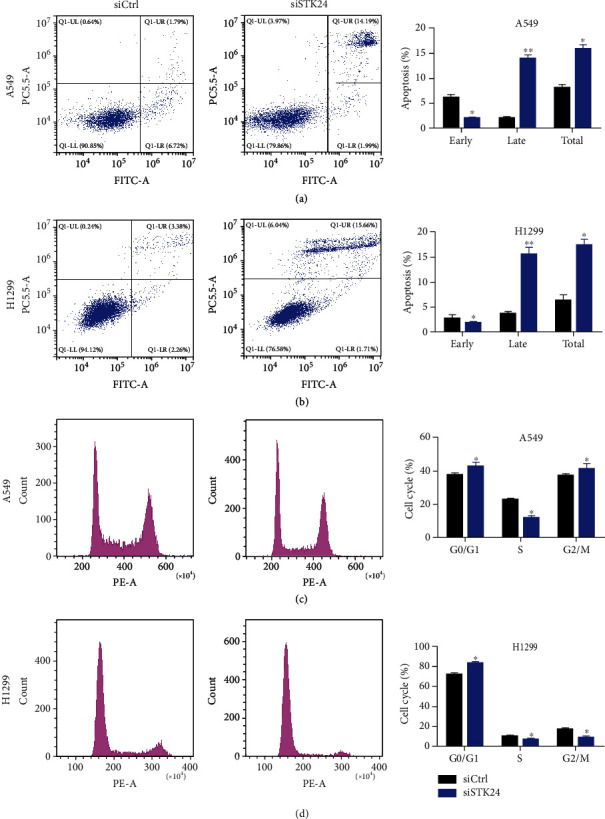
STK24 regulates apoptosis and cell cycle of LUAD. (a, b) Cell apoptosis was detected by PI/annexin V staining. (c, d) Cell cycle was detected by PI staining. ^∗^*p* < 0.05. ^∗∗^*p* < 0.01.

We next investigated whether STK24 regulated cell apoptosis and cell cycle progression by staining the cell with PI/annexin V and PI, respectively. We found that STK24 downregulation resulted in a reduction of early apoptosis but a dramatic enhancement of late apoptosis in the A549 and H1299 cells. Total apoptosis, which included early and late apoptosis, was increased after STK24 knockdown in the cells (Figures 3(a) and 3(b)). Cell cycle analysis found that STK24 downregulation increased the cells at G0/G1 phase but decreased the cells at S phase. Cells at G2/M phase were slightly increased in A549 cells, while they were decreased in H1299 cells (Figures 3(c) and 3(d)). These results generally indicate that STK24 silencing promotes cell apoptosis and cell cycle arrest at G0/G1 phase.

### 3.4. A Positive Regulation between KLF5 and STK24 Exists in Lung Cancer Cells and Patients

**Figure 4:**
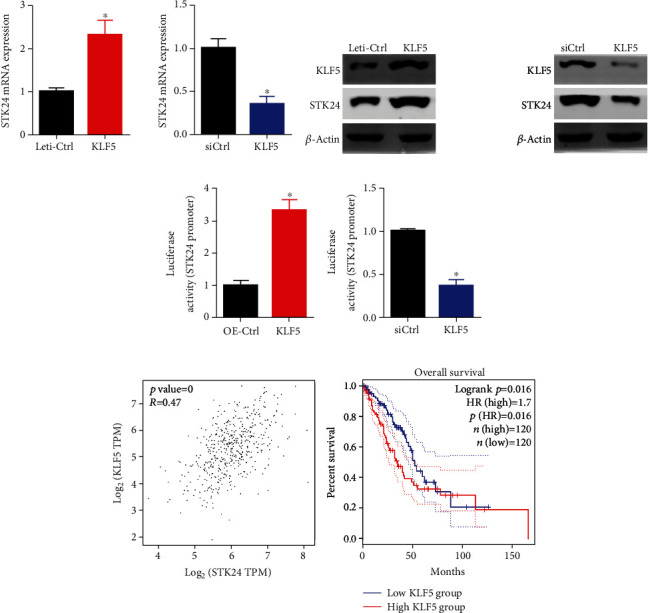
KLF5 promotes the expression of STK24 at transcription level. (a) mRNA expression of STK24 was assessed in KLF5 overexpressed and knockdown A549 cells. (b) Immunoblotting detection of KLF5 and STK24 protein abundance was assessed in KLF5 overexpressed and knockdown A549 cells. (c) Luciferase reporter activity of STK24 promoter was determined in A549 cells after KLF5 overexpression and knockdown. (d) Spearman correlation between KLF5 and STK24 in LUAD samples. Overall survival analysis of LUAD patients who were divided into KLF5 high expression (*n* = 120) and low expression (*n* = 120) groups. ^∗^*p* < 0.05.

KLF5 belongs to the Krüppel-like factor family and has transcription activity. Dysregulation of KLF5 is involved in cancer development. To assess the relationship between KLF5 and STK24, we overexpressed and knocked down KLF5 in A549 cells and checked the expression of STK24. qRT-PCR and immunoblotting results showed that STK24 mRNA and protein expression were upregulated after KLF5 overexpression and downregulated after KLF5 knockdown in A549 cells (Figures 4(a) and 4(b)). Luciferase reporter assay confirmed that KLF5 positively regulated the luciferase activity of STK24 promoter (Figure 4(c)). Analyzing from TCGA data, we found that there was a positive correlation between KLF5 transcript and STK24 transcript in LUAD samples. KLF5 highly expressed patients exhibited shorter overall survival than patients who had low expression of KLF5 (Figure 4(d)). Therefore, KLF5 upregulation of STK24 may contribute to the progression of lung cancer in both cells and patients.

### 3.5. KLF5 Upregulation of STK24 Promotes Lung Cancer Cell Proliferation and Migration

**Figure 5:**
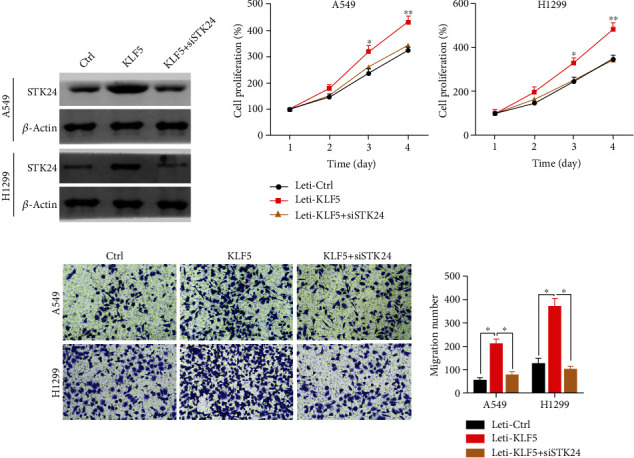
KLF5 promotes cell proliferation and migration through SKT24. (a) Immunoblotting analysis of STK24 in Ctrl, KLF5, KLF5 + siSTK24 A549, and H1299 cells. (b) CCK8 assay was performed in Ctrl, KLF5, KLF5 + siSTK24 A549, and H1299 cells. (c) Cell migration was examined in Ctrl, KLF5, KLF5 + siSTK24 A549, and H1299 cells. ^∗^*p* < 0.05. ^∗∗^*p* < 0.01.

Above results promoted us to further illustrate the function of KLF5/STK24 axis in lung cancer cell function. We then constructed negative control (Ctrl), KLF5 overexpressed (KLF5), and KLF5 overexpressed with silenced STK24 (KLF5 + siSTK24) A549 and H1299 cells. Immunoblotting results confirmed that we successfully constructed the indicated cells (Figure 5(a)). As shown by CCK8 results, we demonstrated that KLF5 overexpression enhanced the proliferation ability of A549 and H1299 cells, which could be reversed by STK24 downregulation (Figure 5(b)). Transwell assay indicated that KLF5 promoted the migration capacity of A549 and H1299 cells, which could also be reduced by STK24 knockdown (Figure 5(c)). Collectively, KLF5 promotes lung cancer cell proliferation and migration and promotes STK24 expression. Inhibition of STK24 expression decreased the ability of KLF5 to promote tumor proliferation and metastasis. KLF5 promotes the proliferation and metastasis of lung cancer cells by promoting the expression of STK24.

### 3.6. STK24 Expression Mediates the Immunomodulatory Function of LUAD

**Figure 6:**
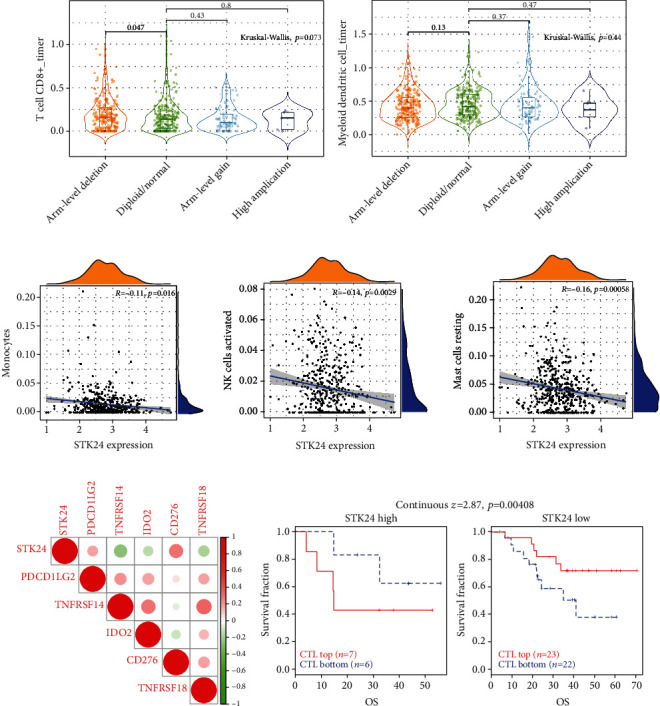
STK24 expression mediates the immunity of LUAD. (a) Relationship between sCNV of STK24 and CD8^+^ T cells. (b) Relationship between sCNV of STK24 and myeloid dendritic cells. (c) The CIBERSORT algorithm analyzed the correlation of STK24 expression with monocytes, activated NK cells and resting mast cells. (d) The relationship between STK24 expression levels and immuneC checkpoints. (e) The TIDE algorithm concluded that different STK24 expression levels and cytotoxic T lymphocyte infiltration weDre associated with LUAD prognosis.

Dysregulation of tumor immune function is a key step in tumorigenesis and development [15]. It has been previously reported that STK24 promotes the expansion of myeloid-derived suppressor cells in gastric cancer. Therefore, in this study, we further explored the relationship between STK24 and tumor immunity. As shown in Figures 6(a) and 6(b), through the analysis of the TIMER database, we found that the deletion of the copy number of STK24 significantly increased the number of CD8 cells, and conversely, the amplification of the copy number of STK24 decreased the number of myeloid dendritic cells. Next, the CIBERSORT algorithm showed that the expression of STK24 was negatively correlated with monocytes, activated NK cells, and resting mast cells (Figure 6(c)). In addition, we further analyzed the relationship between STK24 expression and various immune checkpoints. The results showed that STK24 was positively correlated with PDCD1LG2 and CD276 but negatively correlated with TNFRSF14, IDO2, and TNFRSF18 (Figure 6(d)). Finally, we used the TIDE algorithm to analyze LUAD. The T cell dysfunction score was positive for STK24. Patients with high STK24 expression had poor prognosis and low cytotoxic T lymphocyte infiltration, while patients with low STK24 expression had the opposite prognosis and cytotoxic T lymphocyte infiltration (Figure 6(e)).

## 4. Discussion

LUAD is the most common subtype of lung cancer [16]. In this study, we found that STK24 was highly expressed in LUAD samples based on TCGA data. High expression of STK24 conferred poorer overall and disease-free survival of LUAD patients. Loss-of-function and gain-of-function experiments demonstrated that STK24 expression in lung cancer cells A549 and H1299 was essential to sustain cell growth and proliferation. Cell cycle arrest at G0/G1 and apoptosis were also induced by STK24 knockdown. Thus, STK24 acts as a proliferation inducer for lung cancer.

Protein kinases and phosphatases are important factors in regulating mammals' physiological and pathological functions. Protein kinases promote or suppress the activity of downstream substrate by increasing the phosphorylation of the proteins. The most well-known kinases are PI3K/AKT/mTOR signaling family, the activation of which contributes to the development of a wide variety of malignancies [17–19]. Recently, serine/threonine-protein kinase family attracts oncologists' attention because dysregulation of these proteins participates in cancer development. By knowing that MST1/STK4 mainly functions as a tumor suppressor, while MST2/STK3 can act as an oncogene [20], the role of MST3/STK24 in carcinogenesis should be determined. Although STK24 has been identified as an oncogene in breast cancer [11], it suppresses colon cancer growth [10]. One literature showed that STK24 was highly expressed in LUAD tissues and might be a potential biomarker for LUAD diagnosis [21], whereas the function of STK24 remains to be investigated. Thus, our evidences that STK24 overexpression promoted lung cancer cell proliferation and its knockdown-suppressed cancer cell growth revealed that STK24 acts as an oncogene in lung cancer. We also showed the important role of STK24 in regulating cell cycle progression and apoptosis.

Krüppel-like factor 5 (KLF5) is an important transcription factor. KLF5 overexpression enhances the malignancy of gastric cancer via modulating cell cycle proteins p21 and CDK4 [22]. Overexpression of KLF5 is inversely correlated with the prognosis of colon cancer patients [23]. In prostate cancer, KLF5 interacts with androgen receptor (AR) and contributes to cancer development stimulated by AR signaling [24]. These studies highlight the important role of KLF5 in cancer development. Nevertheless, the downstream effectors of KLF5 need intensive studies. Here, we showed that KLF5 positively regulated the expression of STK24 at transcription level. There was also a positive relationship between KLF5 expression and STK24 expression in LUAD samples. KLF5 overexpression was also inversely correlated with patients' survival. Interestingly, when KLF5 overexpression promoted lung cancer cell proliferation and migration, the knockdown of STK24 significantly blocked the oncogenic role of KLF5.

Immune escape is a key link in tumor metastasis, and changes in the immune microenvironment play a pivotal role in this process [25]. The tumor immune microenvironment is regulated by many factors, such as the tumor itself, and various immune and stromal cells [26]. It has been reported that tumor cells can suppress the immune microenvironment by secreting various cytokines [27]. In this study, we preliminarily found that STK24 has an inhibitory effect on the immune microenvironment by bioinformatics analysis, but we failed to investigate its phenotype and mechanism through in vitro and in vivo experiments. We intend to investigate further in subsequent studies.

In conclusion, KLF5 upregulation of STK24 promotes lung cancer growth and migration. Our findings not only illustrated the important role of STK24 in LUAD but also revealed a possible mechanism that STK24 was upregulated by KLF5 in lung cancer patients. Notably, we also predicted that STK24 might also be involved in the immunomodulatory function of LUAD. Based on these findings, we proposed that targeting STK24 might be a potential therapy for lung cancer patients with highly expressed KLF5.

## Data Availability

The data generated or analyzed in this study are available from the corresponding author for reasonable request.
